# Gut microbiota as a novel target for treating anxiety and depression: from mechanisms to multimodal interventions

**DOI:** 10.3389/fmicb.2025.1664800

**Published:** 2025-10-21

**Authors:** Zhang Ruohan, Wang Ruting, Wu Hongxi, Huang Zhenjin, Liang Jiale, Zhang Rongxin, Jiang Feng, Song Yuanbo

**Affiliations:** ^1^Graduate School, Guangxi University of Chinese Medicine, Nanning, Guangxi, China; ^2^Ruikang Hospital, Guangxi University of Chinese Medicine, Nanning, Guangxi, China

**Keywords:** gut microbiota, depression, anxiety, microbiota-gut-brain axis, intervention

## Abstract

The global prevalence of depression and anxiety continues to rise, with major depressive disorder and anxiety disorders estimated to affect approximately 3.1 and 4.8% of the world’s population. Yet current pharmacological treatments demonstrate limited efficacy. This limitation has spurred extensive research into alternative treatment methods. Emerging evidence highlights a complex correlation between gut microbiota (GM) imbalance and mental health disorders. Disruptions in GM may trigger or exacerbate symptoms of anxiety and depression by interfering with communication pathways between the gut and brain. These pathways include neural signaling through the vagus nerve, hormone regulation via the hypothalamic–pituitary–adrenal (HPA) axis, immune responses involving pro-inflammatory cytokines, and metabolic processes related to short-chain fatty acids (SCFAs). Preclinical studies and initial clinical trials indicate promising results for therapeutic interventions targeting gut microbiota. Given that current evidence remains constrained by insufficient depth of understanding regarding underlying mechanisms, this review explores the intricate interactions among the gut microbiota, and brain, highlighting opportunities for advanced therapeutic approaches, focusing on probiotics, prebiotics, postbiotics, synbiotics, dietary modifications, fecal microbiota transplantation (FMT), fecal virome transplantation (FVT), and traditional Chinese medicine (TCM). It elucidates the role of gut microbiota in depression/anxiety and advances therapeutic approaches.

## Graphical abstract

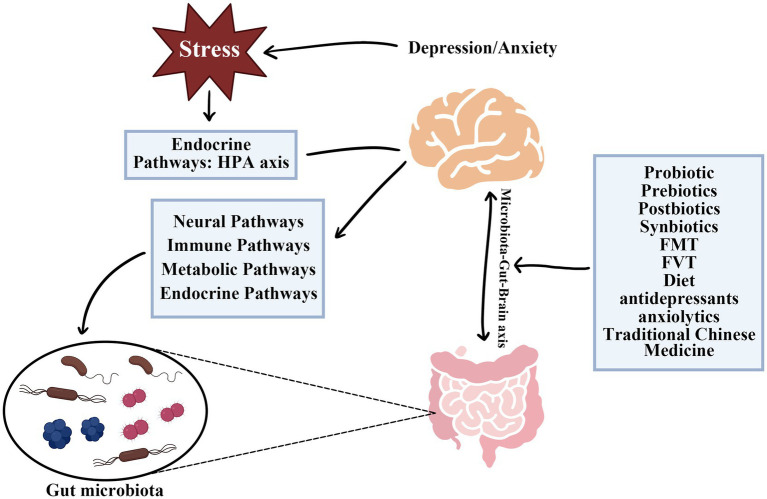

## 1 Introduction

Depression and anxiety are widespread psychological conditions affecting people globally, regardless of age or background (Meier et al., 2016). In recent years, the incidence of mental health disorders, particularly depression, has risen significantly, attracting increasing social attention (Fukuda et al., 2019). In 2020, major depressive disorder and anxiety disorders imposed an extremely heavy global disease burden, with approximately 49.4 million and 44.5 million disability-adjusted life years (DALYs) attributable to depressive disorders and anxiety disorders, respectively. Notably, the COVID-19 pandemic directly contributed to a surge in this burden, adding an estimated 10.7 million and 9.05 million DALYs for these conditions. Approximately 3.15% of the global population suffers from major depressive disorder, while 4.80% experiences anxiety disorders (COVID-19 Mental Disorders Collaborators, 2021). According to a report released by the World Health Organization, the incidence of anxiety disorders and major depressive disorder increased by approximately 26 and 28%, respectively, within just 1 year following the onset of the pandemic (World Health Organization, 2025). These figures underscore the significant impact of mental disorders on population health and emphasize the imperative to strengthen mental health management. Although new pharmacological treatments and psychotherapeutic approaches continue to develop, considerable gaps persist in the treatment landscape (Medina-Rodríguez et al., 2024). Therefore, exploring novel treatment options is necessary to address these issues. Extensive research has highlighted the crucial impact of gut microbiota on the etiology and treatment of depression and anxiety disorders (Chen et al., 2023).

The gut microbiota comprises diverse microbial communities inhabiting the digestive system (Gagnon et al., 2023). As scientific investigation into gut microbiota advances, researchers have discovered that this vast ecosystem of trillions of microorganisms within our digestive tract plays roles extending far beyond digestion. Emerging research demonstrates that these microorganisms significantly influence physical health and mental state (Mayer et al., 2014). Numerous studies have associated gut microbiota composition with the occurrence of anxiety and depression (Ye et al., 2022). Preclinical evidence strongly supports the existence of bidirectional communication among the brain, gastrointestinal system, and gut microbiota (Mayer et al., 2022). Although the exact mechanisms linking the gut and brain in depression remain unclear, there is an increasing awareness of the urgent need to investigate interactions among microbiota, the digestive system, and neural pathways in patients with depression (Chang et al., 2022; Matin and Dadkhah, 2024). By advancing understanding of these interactions, researchers can better develop innovative microbiota-based treatments for depression and anxiety (Liu et al., 2023).

Although extensive research has established the association between the gut microbiota and anxiety disorders and depression, the current knowledge gap in the field is shifting from “whether there is a correlation” to “how it functions” and “how to intervene.” This review aims to synthesize key evidence demonstrating how gut microbiota influence mental health, with a particular emphasis on integrating mechanistic explorations from animal models to human studies. It assesses the existing clinical evidence and translational potential of microbiota-targeted therapeutic strategies, thereby providing a clear theoretical foundation for developing novel microbiome-based treatments for psychiatric disorders.

## 2 The gut microbiota

Microorganisms are abundant in our environment, and even daily activities can influence our microbial communities (Lach et al., 2018). The human gastrointestinal tract harbors a diverse microbial community, including bacteria and fungi, collectively known as the intestinal microbiota. Throughout human evolution, these microorganisms have developed mutually beneficial symbiotic relationships with their hosts. As the host grows, develops, and undergoes physiological changes, the gut microbiota also evolves gradually (Adak and Khan, 2019). The high species diversity of GM provides functional redundancy within the ecosystem, enabling resilience and adaptability to environmental perturbations (Claesson et al., 2012). Specifically, inflammasome activation in patients with depression can exacerbate neuroinflammation, subsequently leading to reduced diversity and imbalance in GM. Therefore, further exploration and confirmation of the interaction between GM composition and these diseases is necessary (Rutsch et al., 2020).

Research indicates an intricate relationship between the GM and its host (Liu et al., 2022), involving numerous key physiological functions (Wang H. et al., 2022). These functions include immune system development and function, food digestion and energy absorption, intestinal endocrine function and nerve signaling, medication metabolism, endotoxin removal, and effects on host mental health (Fan and Pedersen, 2021; Shanahan et al., 2021).

## 3 The microbiota–gut–brain axis

**Figure 1:**
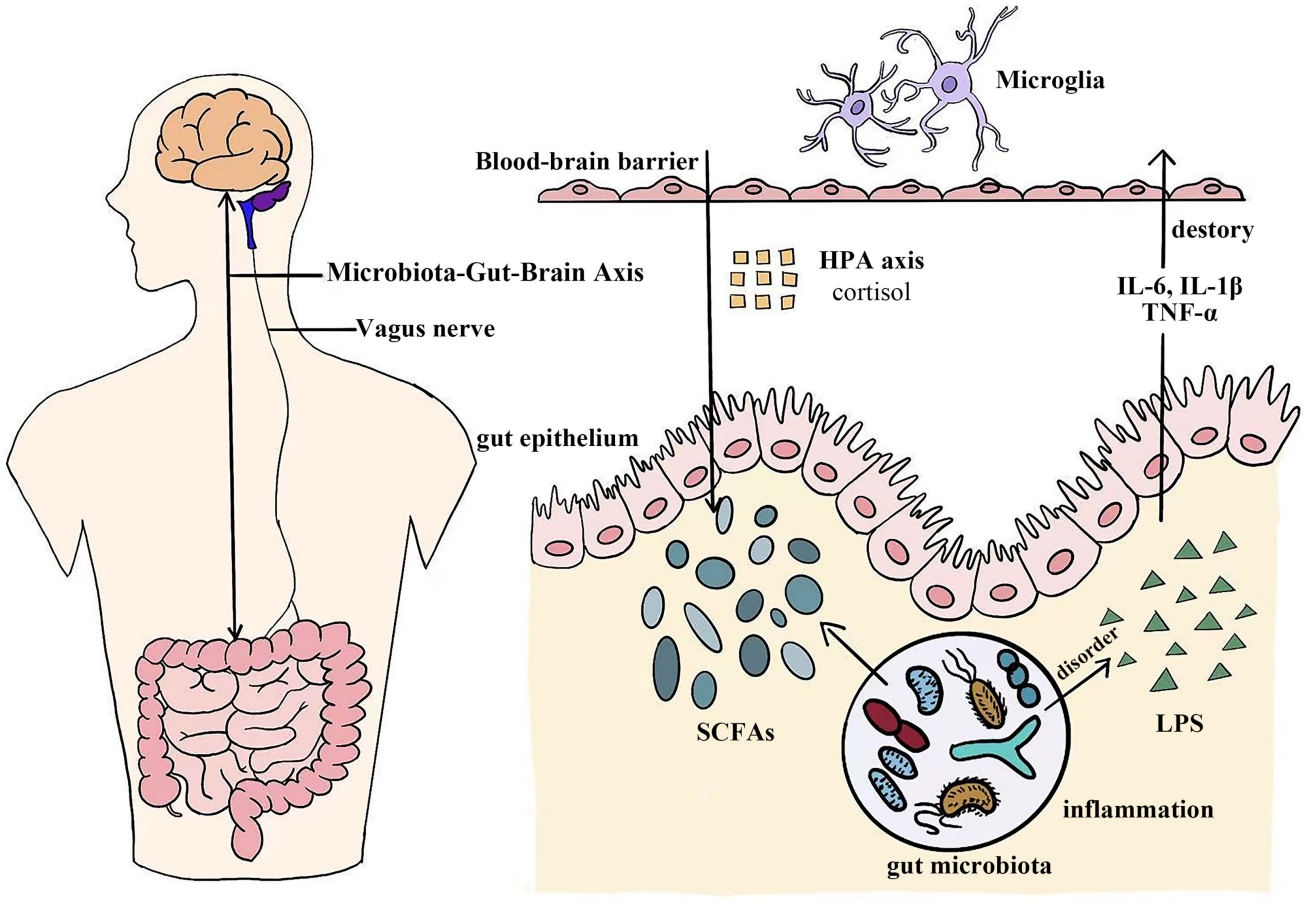
The microbiota-gut-brain axis functions as a sophisticated bidirectional channel connecting the digestive system and the central nervous system. Complex biological pathways, including neural, endocrine, immune, and metabolic pathways, facilitate this dynamic interaction. SCFAs, Short-chain fatty acids; LPS, Lipopolysaccharide; IL-6, Interleukin-6; IL-1β, Interleukin-1β; TNF-*α*, Tumor necrosis factor-α.

The Microbiota-Gut-Brain (MGB) axis is an intricate, bidirectional signaling system dynamically connecting the digestive tract and the brain. This connection significantly influences the functions and homeostasis of both systems (Thomaz et al., 2021). Interaction between intestinal microbiota and the nervous system occurs through multiple barriers, including the intestinal epithelial barrier (IEB), gut vascular barrier (GVB), blood–brain barrier (BBB), plexus vascular barrier (PVB), and blood-cerebrospinal fluid barrier (B-CSF) (Carloni and Rescigno, 2023). Communication within the MGB axis involves dynamic interactions among metabolic, endocrine, neural, and immune systems, highlighting the complexity of gut-brain connections (Góralczyk-Bińkowska et al., 2022). Specific mechanisms of the MGB axis include neural pathways, endocrine pathways (HPA axis regulation), immune pathway, and metabolic pathways (Góralczyk-Bińkowska et al., 2022; Alli et al., 2022; Borkent et al., 2022). Signal transmission in the MGB axis occurs mainly via these neural, endocrine, immune, and metabolic pathways (Dacaya et al., 2025; Figure 1).

### 3.1 Key pathways connecting GM and the brain

#### 3.1.1 Neural pathways

One critical neural pathway is the vagus nerve, which acts as a primary conduit for signal transmission between the gut and brain, influencing emotional regulation and stress responses (Bonaz et al., 2018). Animal studies indicate that vagus nerve stimulation (VNS) effectively alleviates depression, playing a pivotal role in stress modulation and mood regulation (Breit et al., 2018). Research has demonstrated that repetitive VNS (rVNS) produces anxiolytic effects through the noradrenergic pathway. This treatment is associated with *α*-amino-3-hydroxy-5-methyl-4-isoxazolepropionic acid receptor (AMPAR)-mediated excitatory neurotransmission in the central amygdala (CeL), achieved via AMPAR transport activation (Zhang et al., 2022; Toader et al., 2024). Microglia, the primary immune cells of the central nervous system (CNS), initiate immune responses when recognizing neurotransmitter signals (Gao H. et al., 2022; Agirman et al., 2021). Recent studies suggest that gut microbiota in animals experiencing stress activate microglia in the dentate gyrus. This response may interfere with hippocampal neurogenesis and increase depression-related behaviors (He et al., 2024). GM significantly affect brain function through the synthesis of neurotransmitters such as serotonin and gamma-aminobutyric acid (GABA), which are essential for maintaining emotional balance (Dinan and Cryan, 2020). VNS therapy also reduces peripheral blood TNF-*α* levels, which are elevated in depressed patients (Toader et al., 2024; Koopman et al., 2016). Brain-derived neurotrophic factors (BDNF), as a growth factor associated with the gut microbiota, can be influenced by the gut microbiota through neuroendocrine pathways to regulate its expression, thereby modulates hippocampal neurogenesis and contributes to the onset and progression of depression (Han et al., 2025). Thus, neural modulation represents an innovative clinical approach to treating depression and anxiety.

#### 3.1.2 Endocrine pathways

The HPA axis is a central mediator of the body’s stress response and is significantly influenced by the gut microbiota (Bear et al., 2021). The hippocampus plays a key role in regulating the HPA axis, primarily by controlling corticotropin-releasing hormone (CRH) secretion. This effect is mainly exerted by regulating the hypothalamic paraventricular nucleus (PVN), which prompts the anterior pituitary gland to secrete adrenocorticotropic hormone (ACTH). ACTH subsequently induces glucocorticoid (cortisol in humans) secretion from the adrenal cortex (Misiak et al., 2020; Ding et al., 2024). Cortisol, a glucocorticoid produced by the adrenal cortex (Rehman et al., 2022), increases in response to gut microbiota alterations affecting the HPA axis (Misiak et al., 2020). These hormones significantly influence anxiety and depression (Martin et al., 2019; Sun et al., 2022). Chronic stress negatively affects gut microbiota composition, elevating cortisol levels and exacerbating anxiety and depression. This cascade results from dysfunction of the HPA axis, disturbing gut bacterial balance (Hassamal, 2023). Additionally, gut microbiota modulates neuropeptide and hormone secretion, including mood-regulating hormones such as ghrelin and leptin (Zheng et al., 2016).

#### 3.1.3 Immune pathways

The gut microbiota in healthy individuals typically remains balanced and stable. When pathogens invade and disrupt host-microbial homeostasis, dysbiosis occurs, increasing intestinal barrier permeability (“leaky gut”). This increased permeability allows microbial metabolites, toxins, and pathogens to enter circulation, triggering systemic inflammation (Alhasson et al., 2017; Kociszewska and Vlajkovic, 2022). Intestinal permeability is intricately linked to systemic inflammation and psychiatric disorders (Ma et al., 2022). The enteric nervous system (ENS) plays a crucial role in modulating intestinal immune functions by mediating associated immune responses (Lu et al., 2024). Further research indicates that immune responses are closely associated with neuroinflammation, a major contributor to mood disorders (Sittipo et al., 2022). During inflammatory responses, the body produces numerous pro-inflammatory cytokines. These cytokines, upon entering the brain, modulate neural circuits and neurotransmitter systems related to mood, potentially causing depressive symptoms (Zhu R. et al., 2024). Disruption of gut microbiota also increases lipopolysaccharide (LPS) production (Li W. et al., 2020). LPS-induced inflammation can provoke depressive symptoms through the release of pro-inflammatory cytokines and inflammatory mediators (Shi et al., 2019). Consequently, LPS exacerbates inflammation and disrupts the blood–brain barrier (Logsdon et al., 2020). LPS and similar molecules activate immune cells to release pro-inflammatory cytokines (e.g., IL-6, IL-1β, TNF-*α*), which directly contribute to depression onset (Medina-Rodriguez et al., 2018; Simpson et al., 2021). Additionally, GM imbalance and HPA axis dysfunction mutually influence each other, jointly promoting inflammation (Warren et al., 2024). Microglia, the primary resident macrophages of the CNS, play a key role in brain immune defense (Thangaraj et al., 2018). The gut microbiota may impact microglia via inflammatory signaling along the MGB axis, affecting brain function and modulating depressive-like behaviors (Agirman et al., 2021; Donoso et al., 2023). Studies indicate an increased prevalence of depression and anxiety among patients with ulcerative colitis (UC). Preventive anti-inflammatory treatment effectively alleviates depressive behaviors and reduces systemic inflammatory cytokine levels in colitis models (Yuan et al., 2021).

#### 3.1.4 Metabolic pathways

Key metabolites of the intestinal microbiota, SCFAs, including butyrate, acetate, and propionate (Dong et al., 2022). SCFAs can cross the BBB, providing neuroprotection by regulating neuroinflammation and enhancing barrier integrity (Dalile et al., 2019). SCFAs predominantly inhibit histone deacetylase (HDAC) activity, thereby dampening inflammation and mitigating overzealous immune reactions in microglia, or triggering free fatty acid receptors (e.g., FFAR2/3), to augment microglial activation (Cao et al., 2025). Human trials indicate that an almond-based low-carbohydrate diet (a-LCD) promotes the growth of SCFA-producing gut microbiota, thereby increasing SCFA production, activating the GPR43 receptor, and enhancing GLP-1 secretion to alleviate depressive symptoms (Ren et al., 2020). Butyrate is essential for maintaining intestinal barrier function in mammals. Reduced butyrate levels may trigger depressive symptoms by disrupting gut-derived metabolite balance and altering the expression of G protein-coupled receptors (GPCRs) and BDNF in the CNS (Nohesara et al., 2023). Butyrate stimulates BDNF secretion (Romo-Araiza et al., 2018), a gene potentially associated with anxiety and depression (Chu et al., 2022). Research indicates that sodium butyrate alters gene expression in hippocampal microglia, thereby eliminating lipopolysaccharide-induced depressive-like behavior (Yamawaki et al., 2018). Valeric acid, another gut microbial metabolite, influences MGB axis metabolism (Lin et al., 2021), has demonstrated potential to modulate anxiety and depression (Mann et al., 2014; Bhandage et al., 2019). This finding offers new insights into treating related neurological conditions. Moreover, GM significantly contributes to tryptophan metabolism (Zhao et al., 2022), a crucial precursor of serotonin (Emmerzaal et al., 2020), which profoundly affects mood and emotional regulation (Li Z. et al., 2020). During inflammatory states, tryptophan is metabolized by IDO1 (indoleamine 2,3-dioxygenase) into kynurenine, simultaneously producing neurotoxic metabolites that reduce 5-HT synthesis and promote depression and anxiety (Tahiri et al., 2024). Research indicates that patients who orally administered the probiotic *B. breve* CCFM1025 exhibited a significant increase in fecal concentrations of multiple tryptophan derivatives, such as 5-hydroxytryptophan (5-HTP). These changes in derivative concentrations were significantly correlated with improvements in patients’ depressive symptoms and gastrointestinal symptoms (Tian et al., 2022). Another metabolite, bile acids, showed in randomized controlled trials that elevated glycine-conjugated bile acids were significantly associated with decreased anxiety scores (HADA) (Martin et al., 2024). A study has demonstrated for the first time in humans that the probiotic strain LP299v can significantly reduce plasma homocysteine levels by modulating the tryptophan-homocysteine metabolic pathway. This reduction in homocysteine levels was significantly associated with improvements in cognitive function (Rudzki et al., 2019). Gut peptides are peptide hormones capable of regulating gastrointestinal motility through brain-gut axis interactions (Li et al., 2015; Młynarska et al., 2022). Certain intestinal peptides, including peptide YY (PYY), glucagon-like peptide-1 (GLP-1), cholecystokinin, CRH, growth inhibitory-releasing peptide, and oxytocin, are especially important, as they modulate depressive and anxiety-related behaviors (Młynarska et al., 2022; Dockray, 2014).

**Figure 2:**
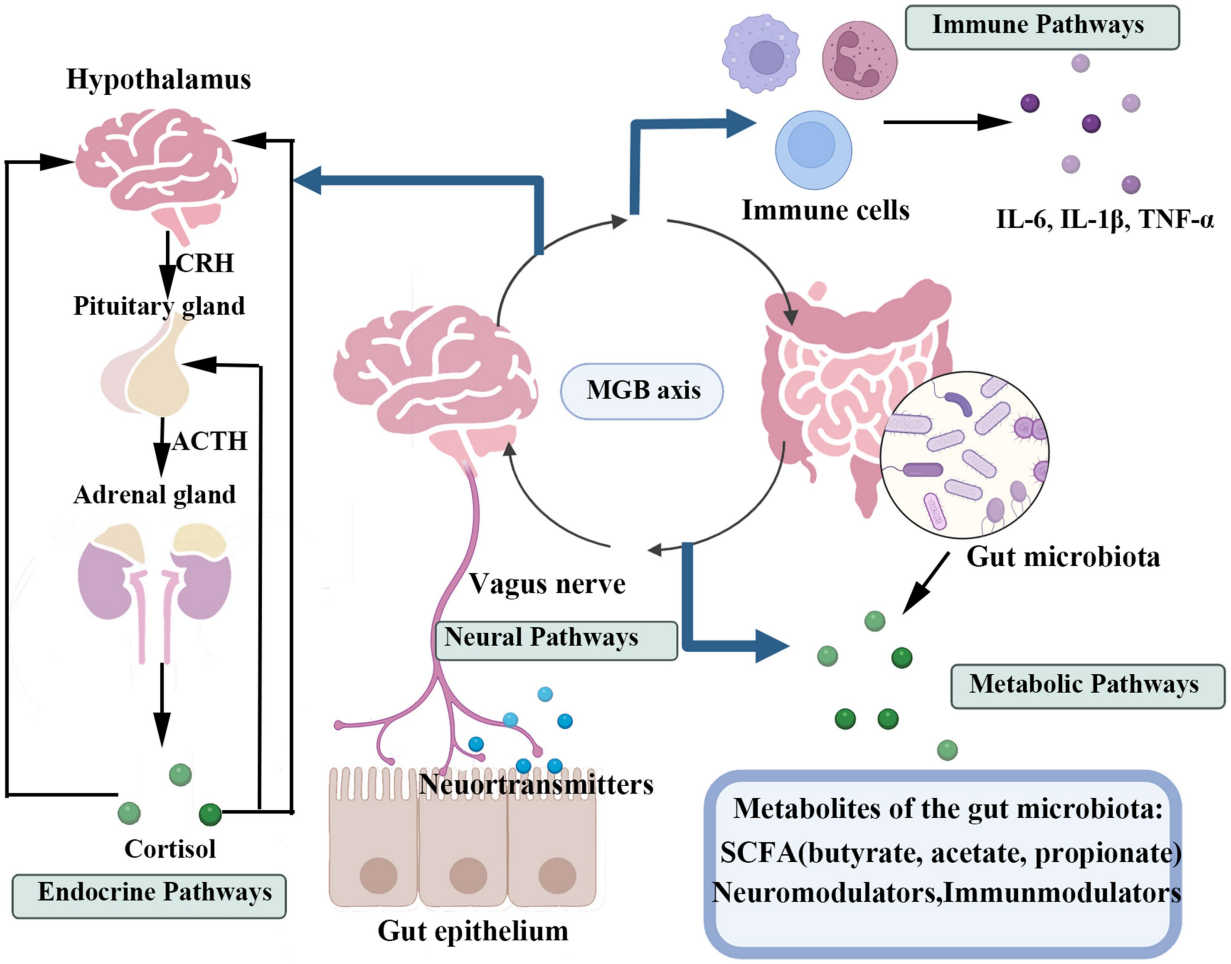
This figure illustrates the primary pathways of bidirectional communication between the gastrointestinal tract and the central nervous system, the GMB axis. The four key pathways include: The neural pathway, primarily mediated by the vagus nerve; The endocrine pathway, involving gut hormones and the HPA axis; The immune pathway, mediated by inflammatory responses and immune cells; And the metabolic pathway, driven by metabolites produced by the gut microbiota (such as SCFAs).

The MGB axis functions as a dynamic hub, through which gut microbial communities communicate via multiple signaling pathways (Figure 2), significantly influencing mental health. Clarifying these relationships is essential for developing novel therapeutic approaches to mental health disorders through GM modulation (Shoubridge et al., 2022).

## 4 Composition and diversity of gut microbiota in anxiety and depression

The intestinal flora of healthy individuals predominantly includes *Bacteroidetes*, *Firmicutes*, *Actinobacteria*, and *Proteobacteria*, collectively comprising approximately 99% of the microbial community (Guo et al., 2022; Lee and Chang, 2021). These phyla are essential for maintaining the physiological and psychological balance of the host (Chen et al., 2022; Valdes et al., 2018). A diverse and stable gut microbiota is crucial for intestinal health, immune responses, nutrient assimilation, and mood modulation via the GMB axis (Cryan et al., 2020).

However, individuals with anxiety and depression frequently display significant shifts in GM composition (Barandouzi et al., 2020; Shen et al., 2021). Recent studies comparing intestinal microbial characteristics between patients with depression and healthy individuals revealed significant differences in microbial composition. Nikolova et al. identified a cross-diagnostic commonality present in psychiatric disorders such as major depressive disorder and anxiety disorders, characterized by a reduction in anti-inflammatory, butyrate-producing genera (e.g., *Faecalibacterium* and *Coprococcus*) and increased pro-inflammatory genera (e.g., *Eggerthella*) (Nikolova et al., 2021). This finding suggests a potential shared microbial. Extensive research has documented reduced microbial diversity, especially alpha diversity, associated with diminished stress resilience and increased susceptibility to mood disorders (Knight et al., 2023). Emerging research consistently identifies decreased abundance of beneficial bacteria, particularly *Lactobacillus* and *Bifidobacterium*, as characteristic features in individuals with mood disorders (Aizawa et al., 2018). Studies demonstrate that *Lactobacillus* and *Bifidobacterium* species produce GABA and serotonin (5-hydroxytryptamine), crucial neurotransmitters involved in mood regulation (Kaur et al., 2023). Further research indicates that depletion of these beneficial bacteria can disrupt neurotransmitter synthesis, influencing both gut and brain functions and significantly impacting mental health (Huang et al., 2020).

Specific microbial taxa have been associated with anxiety and depression. For example, elevated levels of pro-inflammatory bacteria from genera such as Escherichia and Enterobacter have been linked to increased systemic and neuroinflammatory responses. These inflammatory responses potentially contribute to the underlying pathophysiology of mood disorders (Zheng et al., 2016; Simpson et al., 2021). Individuals with depression often exhibit lower levels of SCFA-producing bacteria, indicating a potential protective role against depressive symptoms (Cheng et al., 2024).

Studies involving animal and human subjects suggest an association between gut microbial diversity and mental health, indicating that microbiota disturbances may contribute to depression onset (Shoubridge et al., 2022; Zhu et al., 2020). Yaoyong Lai’s study employed a bidirectional two-sample Mendelian randomization analysis, revealing that gut microbiota dysbiosis is a causative factor in depression and anxiety, rather than merely a consequence of these disorders (Lai and Xiong, 2025). Decreased gut microbial diversity may impair enteroendocrine cell (EEC) activity and disrupt peptide secretion. Such disturbances could affect emotional states and behavior via gut-brain interactions, increasing vulnerability to anxiety and depression (Lach et al., 2018). Dietary interventions represent promising strategies to modify diet components, alleviate depressive symptoms, promote healthier gut microbiota, and enhance beneficial microbial populations (Dai et al., 2025). Probiotics (e.g., *Lactobacillus* and *Bifidobacterium*) can reverse antibiotic-induced alterations in gut microbiota and improve cognitive function and memory through the MGB axis. Thus, probiotics may help restore gut microbial diversity and enhance mental health (Hao et al., 2020). These findings suggest that regulating gut microbiota through certain interventions could alleviate anxiety and depression.

## 5 Therapeutic interventions targeting gut microbiota

Recent studies conducted within the past 5 years have explored the correlation between anxiety, depression, and gut microbiota, as well as possible therapeutic approaches. For example, research suggests that Enterobacteriaceae bacteria may significantly influence mental health, although detailed investigations into the mechanisms remain ongoing. Preliminary experimental results indicate that colibactin-producing *Escherichia coli* (CoPEC), as part of the gut microbiota, may disrupt the microbiota-gut-brain (MGB) axis (Rondepierre et al., 2024). Experimental findings by Xu et al. (2024) suggest that NLRP3 inflammasomes are key components of the MGB axis, indicating that targeting the gut microbiota could become a novel treatment strategy for postpartum depression (PPD). Ongoing research involves numerous studies evaluating the potential effectiveness of gut microbiota regulation in treating depression.

**Figure 3:**
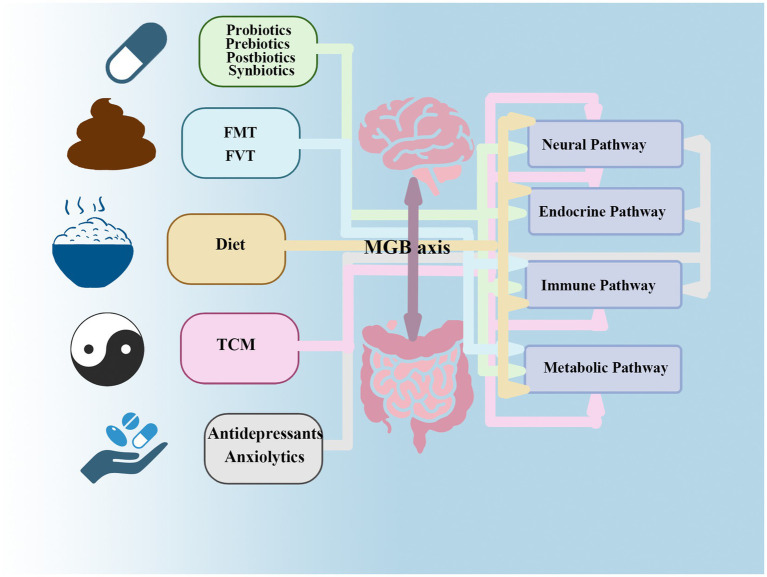
This figure reveals that gut microbiota interventions targeting anxiety and depression likely exert effects through multi-pathway mechanisms of the MGB axis. While certain therapies have primary sites of action (e.g., antidepressants acting on neural synapses), they also regulate endocrine (HPA axis) and immune (inflammatory) pathways. Interventions initiated in the intestinal lumen (e.g., dietary modifications) transmit signals upward through immune, endocrine, metabolic, and neural pathways, thereby achieving therapeutic effects at the neurological and psychological levels. FMT primarily acts by rapidly improving metabolic and immune pathways, subsequently triggering positive chain reactions in endocrine and neural pathways.

Collectively, evidence elucidating the mechanisms by which gut microbiota influence mental disorders underscores their therapeutic potential in managing depression and anxiety, opening promising avenues for addressing mental health issues (Ancona et al., 2021). The therapeutic strategies outlined below(Table 1)—including probiotics, prebiotics, postbiotics, synbiotics, dietary interventions, FMT, FVT, and TCM all leverage this mechanistic link (Figure 3).

**Table 1:** Overview of gut microbiota-targeted interventions for anxiety and depression: clinical and preclincal studies.

Therapeutic interventions	Specific formulation	Dosages and regimen	Study designs	Clinical outcomes	Reference
Probiotics	*Bifidobacterium breve 207–1*	Low-dose:1 × 10^10^ CFU/day; high-dose: 5 × 10^10^ CFU/day; 28 days	RCT	GABA levels were elevated, HPA axis hormones were generally suppressed, 5-HT levels showed no significant change, and mood scale scores remained largely unchanged	Li et al. (2024)
Prebiotics	Galactooligosaccharide	5 g/day; 8 weeks	RCT	No significant effect has been observed	Kazemi et al. (2019)
Postbiotics	the CFS obtained from *Lactobacillus rhamnosus UBLR-58* and *Bifidobacterium breve UBBr-01*	CFS (1 mL/rat); 7 days	Preclincal	Significant improvement in neurological function	Rahman et al. (2024)
Synbiotics	prebiotics, probiotic containing *Lactobacillus acidophilus T16*, *Bifidobacterium bifidum BIA-6*, *Bifidobacterium lactis BIA-7*, and *Bifidobacterium longum BIA-8*	15 g of prebiotics, 5 g of probiotic containing *Lactobacillus acidophilus T16*, *Bifidobacterium bifidum BIA-6*, *Bifidobacterium lactis BIA-7*, and *Bifidobacterium longum BIA-8;*2.7 × 10^7^ CFU/g each; 12 weeks	RCT	Supplementing with Synbiotic can improve depressive symptoms and serum BDNF levels in HD patients	Haghighat et al. (2021)
FMT	Enterobacter FMT capsules	30 tablets every other day for a total of 3 doses;12 weeks	RCT	IBS-SSS scores, anxiety and depression scores decreased significantly	Guo et al. (2021)
FVT	NA	NA	NA	NA	NA
Diet	Mediterranean diet	NA	RCT	The Mediterranean diet has no effect on depression scores	Sharifi et al. (2025)
Antidepressants	Venlafaxine	75 mg/d; the maximal dose was 225 mg/d; 8 weeks	RCT	SSRIs are effective in treating severe depression in postmenopausal women	Zhou et al. (2021)
Anxiolytics	Fluoxetine	20 mg/day, 12 weeks	A pilot prospective randomized open blinded endpoint (PROBE) study	It has a beneficial effect on patients with mild post-stroke anxiety.	Barki et al. (2025)
Traditional Chinese Meicine	Xiaoyaosan	1 mL/100 g body weight, 4 weeks	Preclincal	Exercises antidepressant effects by regulating the “microbiome-gut-brain” axis	Liu et al. (2024)

NA, represents no data.

### 5.1 Microbiota-targeted biologics

#### 5.1.1 Probiotics

Given the correlation between gut microbiota and anxiety/depression, probiotics may alleviate symptoms by regulating dysbiosis (Jang et al., 2019). Probiotics, as a safe and well-tolerated non-pharmacological therapy, represent a promising therapeutic option for mood disorders (Merkouris et al., 2024). Research has shown that probiotics can enhance cognitive function and reduce depressive symptoms, suggesting their potential role in depression treatment (He et al., 2023). Timothy G et al. defined the concept of psycholbiotics as live microorganisms that, when ingested in sufficient quantities, confer health benefits to individuals with mental disorders. These probiotics belong to a class capable of producing and delivering neuroactive substances such as GABA, serotonin, and dopamine, thereby influencing brain function through the MGB axis (Dinan et al., 2013). Probiotic preparations commonly used in clinical practice include beneficial strains such as *Lactobacillus casei*, *Lactobacillus acidophilus*, and *Bifidobacteria* (Zhang et al., 2023). Research by Kirsten Tillisch et al. has demonstrated for the first time in humans that chronic consumption of fermented dairy products containing five probiotic strains—including *Bifidobacterium lactis CNCM I-2494*—significantly modulates the brain’s response to emotional stimuliation (Tillisch et al., 2013). In addition, Gu et al. (2020) confirmed that *Lactobacillus casei* exert significant antidepressant effects in rat models by regulating gut microbiota and the brain-derived neurotrophic factor-tyrosine kinase receptor (BDNF–TrkB) signaling pathway. Certain strains, such as *Lactobacillus NK41*, *Bifidobacterium longum NK46*, and their combinations, can alleviate stress-induced anxiety/depression-like behaviors. These effects involve reduced neuroinflammation (e.g., hippocampal NF-κB and microglial activation), increased neuroplasticity markers (e.g., BDNF), and improved peripheral stress/inflammation indicators (e.g., cortisol, TNF-*α*, and IL-6) (Han and Kim, 2019). Similarly, Sara Ferrari reported that probiotic combinations (e.g., *Bifidobacterium longum novaBLG2*) can mitigate glutamate-induced anxiety/depression symptoms and enhance cell survival by regulating gut-brain axis metabolites (Ferrari et al., 2024). Research also indicates that daily supplementation with *Bifidobacterium breve Bif11* for 21 consecutive days can prevent LPS-induced depressive behavior in mice (Sushma et al., 2023). *Bifidobacterium bifidum* has demonstrated positive preventive and therapeutic effects on depression in animal models and humans (Li et al., 2023). Regarding dosage, studies have indicated that psychobiotics require a minimum daily dose of 1 billion CFU, with optimal results achieved using doses exceeding 10 billion CFU for at least 8 weeks (Jach et al., 2023). The following are partial experimental results from human studies (Table 2).

**Table 2:** Probiotics in human randomized controlled trials.

Study designs	Probiotics	Interventions	Outcomes	References
A randomized, double-blind, placebo-controlled trial. 120 adults experiencing high levels of mental stress	*Bifidobacterium breve 207–1*	Low-dose:1 × 10^10^ CFU/day; high-dose: 5 × 10^10^ CFU/day; 28 days	GABA levels were elevated, HPA axis hormones were generally suppressed, 5-HT levels showed no significant change, and mood scale scores remained largely unchanged	Li J. et al. (2024)
A randomized, double-blind, placebo-controlled trial. 43 patients with major depressive disorder	*Bifidobacterium lactis W51*, *B. lactis W52*, *Lactobacillus acidophilus W22*, *L. casei W56*, *L. paracasei W20*, *L. plantarum W62*, *L*. *salivarius W24*, *L. lactis W19*	1 sachet, 3 g twice daily, 1.5 × 10^10^ CFU in total; 3 months	Significantly alters vagal nerve function and gut microbiome composition in patients with major depressive disorder	Mörkl et al. (2025)
A randomized, double-blind, placebo-controlled trial. 266 patients with anxiety and depression following gastrointestinal cancer surgery	*Bifidobacterium animalis* subsp., *Bifidobacterium breve* DSM 16604, *Bifidobacterium longum* DSM 16603, *Lacticaseibacillus rhamnosus* ATCC 53103	4 sachets, twice daily; 4 weeks	Probiotic therapy benefits cancer patients by alleviating anxiety, depression, and other psychological symptoms following surgery.	Tzikos et al. (2025)
A randomized, multicenter, parallel-group clinical trial. 34 patients undergoing Hemodialysis	*Bifidobacterium breve CNCM I-4035*, *Bifidobacterium animalis lactis CECT 8145*, *Lactobacillus paracasei CNCM I-4034*	5 × 10^9^ CFU/day; 6 months	Significantly improves depressive symptoms and quality of life, stabilizes plasma LPS levels, and modulates gut microbiota composition.	Climent et al. (2025)
A randomized, multicenter, parallel-group clinical trial. 50 patients with major depressive disorder	*Bacillus subtilis*, *Bifidobacterium bifidum*, *B. breve*, *B. infantis*, *B*. *longum*, L. *acidophilus*, L. *delbrueckii* ssp. *Bulgaricus*, L. *casei*, L. *plantarum*, L. *rhamnosus*, L. *helveticus*, L. *salivarius*, *Lactococcus lactis*, *Streptococcus thermophilus*	4 capsules/day (2 × 10^9^ CFU/capsule); 8 weeks	Significantly reduces depression and anxiety scores in patients	Nikolova et al. (2025)
A randomized, double-blind, placebo-controlled trial. 67 overweight elderly individuals with anxiety and depression	*Lactobacillus helveticus* R0052, *Bifidobacterium longum R0175*	3 × 10^9^ CFU/day;8 weeks	A positive effect on alleviating depressive symptoms in this population.	Noori et al. (2025)

In recent years, multiple meta-analyses have attempted to comprehensively evaluate the efficacy of probiotics for mood disorders. For example, Liu’s analysis revealed no statistically significant difference between the probiotic group and the placebo group in alleviating anxiety symptoms (SMD = −0.12, 95% CI: −0.28 to 0.04, *p* = 0.14; Liu et al., 2018). However, Moshfeghinia et al. (2025) analysis indicates that probiotics, prebiotics, or synbiotics can significantly alleviate depressive and anxiety symptoms in individuals with depression, despite high heterogeneity among studies (*I*^2^: 96.29%). Notably, nearly all meta-analyses emphasize significant heterogeneity among included studies (*I*^2^ > 50%), underscoring the need for extreme caution in interpreting results.

Despite these promising findings, probiotic applications still face several limitations. Although evidence supports probiotics’ benefits for mood regulation, specific biological mechanisms remain unclear. Additionally, probiotic efficacy varies among individuals, likely due to differences in gut microbiota composition, lifestyle, and genetic background. Therefore, considering that the gut microbial composition of individuals with mental illness differs from that of healthy individuals, personalized intervention strategies can be designed to address specific microbial dysbiosis for therapeutic purposes (Vindegaard et al., 2021). Furthermore, probiotics used in different studies vary in strain, dosage, and administration protocols, reducing comparability across studies. Crucially, the effects of probiotics exhibit a high degree of strain specificity. This means that different strains-even of the same species-may have completely different effects on their hosts. For example, experiments have demonstrated that *Lactobacillus johnsonii* (NCC 533) or *Lactobacillus paracasei* (NCC 2461)—two strains of intestinal *lactobacilli* exhibiting similar characteristics *in vitro*—can elicit different immune responses (Ibnou-Zekri et al., 2003). Even the same bacterial strains exhibit differences in dosage and treatment duration across different experiments. Therefore, it is unscientific to summarize the results of probiotic research in general. Future research and clinical applications must operate accurately at the strain level. Although some studies have shown positive effects, research in this field is extremely heterogeneous, resulting in different studies often reaching conflicting conclusions. This heterogeneity mainly comes from inconsistent intervention plans, different study populations, diverse outcome measures, and lack of standardization.

#### 5.1.2 Prebiotics

Prebiotics are substrates selectively utilized by host microorganisms to confer health benefits (Gibson et al., 2017). Non-digestible oligosaccharides, including fructan, galactan, and resistant starch, are the primary prebiotic components. Recently, the scope of the term has broadened to include dietary compounds such as polyphenols (Medina-Larqué et al., 2022). Prebiotics affect depression by modulating neurotransmitter production, SCFA production, and immune regulation (Yang Y. et al., 2023). SCFA production is closely linked to prebiotic intake and the composition of intestinal microbiota (Godínez-Méndez et al., 2021). SCFAs can cross the BBB, influencing brain function (Wang J. et al., 2022). Additionally, studies suggest that SCFAs regulate emotional states through the MGB axis and produce anti-inflammatory effects (Liu et al., 2021; Van de Wouw et al., 2018). Recently, Igor Henrique Rodrigues de Paiva succeeded in reversing depressive and anxiety symptoms in mice fed a high-fat diet through therapeutic intervention with fructooligosaccharides (FOS) and galactooligosaccharides (GOS) (Paiva et al., 2024). The administration of FOS and GOS not only demonstrated benefits in probiotic treatment for stress-related behaviors but also effectively reduced stress-induced increases in corticosterone and pro-inflammatory cytokines, attenuating depressive-like and anxiety-like behaviors (Burokas et al., 2017). Antonio Leo’s research confirmed that in the chronic unpredictable mild stress (CUMS) model, the combination of prebiotic *α*-lactalbumin (ALAC) and postbiotic sodium butyrate (NaB) effectively regulates the MGB axis. This synergy indicates potential for treating anxiety and depression (Leo et al., 2021). However, clinical data on prebiotics remain scarce. Thus, large-scale clinical trials are necessary to determine their sustained therapeutic effects, optimal dosage, and mechanisms of action against depression and anxiety (Asad et al., 2025).

#### 5.1.3 Postbiotics

The International Scientific Association for Probiotics and Prebiotics (ISAPP) defines postbiotics as formulations containing non-viable microorganisms or their constituents that provide health benefits to the host (Salminen et al., 2021). Given the limitations associated with probiotic research, postbiotic compositions derived from probiotics may represent promising new microbial therapeutic approaches (Nataraj et al., 2020; Suez et al., 2019). Postbiotics comprise inactivated cell components (e.g., cell wall fragments and surface proteins) and bacterial cell metabolites (e.g., SCFAs and bacteriocins), collectively mediating physiological effects (Mehta et al., 2023). Postbiotics have been employed to regulate intestinal microbiota, showing effectiveness in resolving dysbiosis (Gao M. et al., 2022), which may positively impact mental health. A review by Icer et al. (2024) noted that GABA produced by lactic acid bacteria (LAB) may exert neuroprotective effects, improving depression. Another study reported that cell-free supernatant (CFS) obtained from *Lactobacillus rhamnosus UBLR-58* and *Bifidobacterium breve UBBr-01* can reduce nerve damage by inhibiting neuroinflammation and regulating the MGB axis pathway (Rahman et al., 2024). Additionally, SCFAs produced as postbiotics by probiotics play an important role in the MGB axis (Ragavan and Hemalatha, 2024). Increasing research into postbiotics and neurological dysfunction disorders suggests that modulation of the gut-brain axis offers promising future applications in mental health.

#### 5.1.4 Synbiotics

Synbiotics, dietary supplements combining probiotics and prebiotics to deliver synergistic health benefits, demonstrate therapeutic potential in mood disorders (Ke et al., 2019). A 2020 double-blind randomized controlled trial (RCT) showed that Synbiotic 2000 supplements can improve emotional regulation in adults with attention deficit hyperactivity disorder (ADHD) (Skott et al., 2020; Arteaga-Henríquez et al., 2024). A multicenter, randomized, placebo-controlled “basket” trial by Arteaga-Henríquez et al. (2024) also demonstrated that synbiotics specifically improved mood symptoms and abnormalities in mood regulation, consistent with Scott’s findings. A preclinical mechanistic study by Lapmanee et al. (2024) revealed that synbiotics have neuroprotective and gut benefits in stressed rats. Sanjay Noonan et al. found that supplementation with Synbiotic produced statistically significant improvements in measures of depression (Noonan et al., 2020). Synthetic probiotics possess both probiotic and prebiotic properties, and combining these elements primarily enhances probiotic survival during gastrointestinal transit. When properly combined, they may produce better results than probiotics or prebiotics alone (Yoo et al., 2024; Markowiak and Śliżewska, 2018).

### 5.2 Microbiota transplantation therapies

#### 5.2.1 Fecal microbiota transplantation

Fecal microbiota transplantation (FMT) is a therapeutic intervention involving the transfer of fecal material from a healthy individual to the intestinal tract of a recipient. This approach has recently gained attention as a potential treatment for depressive disorders by recalibrating intestinal microbiota (Joo et al., 2024). Multiple academic analyses indicate that FMT interventions reduce bacteria associated with psychiatric disorders (e.g., *Lactobacillus acidophilus*), thereby alleviating psychiatric symptoms (Yang C. et al., 2023). A recent RCT of patients with diarrhea-predominant irritable bowel syndrome (IBS-D) and comorbid anxiety/depression demonstrated that oral FMT capsules significantly alleviated both IBS-D and associated psychological symptoms (Guo et al., 2021). Critically, these clinical improvements were accompanied by the restoration of gut microbial communities, suggesting microbiota modulation as a potential underlying mechanism. A pilot RCT by Green et al. (2023) demonstrated the feasibility of a different FMT administration route (enema) for patients with major depressive disorder (MDD). Baske et al. (2024) reviewed evidence showing significantly elevated Escherichia-Shigella levels in patients with generalized anxiety disorder (GAD) and proposed that FMT might reduce these bacteria, positively affecting the gut-brain axis and providing a promising avenue for novel GAD therapies. Xu Qi et al. showed that FMT from healthy mice to postpartum depression (PPD) model mice improved depression/anxiety-like behaviors by regulating the NLRP-3/caspase-1 pathway in the gut and hippocampus (Campbell and Macqueen, 2004). Cai et al. (2022) confirmed that FMT improved CUMS-induced depressive behavior in rats, with mechanisms involving coordinated actions of the 5-HT system, BDNF, neurotransmitters (GABA/Glu), inflammatory factors (IL-6/LPS), and SCFAs. Rao et al. (2021) observed increased 5-HT and decreased IL-1β and TNF-*α* after FMT treatment, leading to improved depressive symptoms. An international consensus meeting convened by the American Gastroenterological Association (AGA) indicated that FMT demonstrates favorable short-term safety in treating *Clostridium difficile* infection, but long-term safety data remain insufficient and require ongoing follow-up studies (Cammarota et al., 2019). Moreover, evidence is lacking regarding its safety in extremely immunocompromised individuals (Yadegar et al., 2024). Mark Hofmeister’s recent meta-analysis ultimately included 62 studies, with only one evaluating the impact of FMT on depressive symptoms. The conclusion was that this intervention did not demonstrate a statistically significant benefit for depression (Hofmeister et al., 2021). However, the author has only included one relevant study thus far. Due to factors such as small sample size, it is currently not possible to draw definitive conclusions on this matter.

#### 5.2.2 Fecal virome transplantation

The goal shared by FMT and FVT is to treat diseases by reshaping gut microbiota. Mao et al. (2024) suggested that the success of FMT may largely be attributed to the role played by its transferred viral components in reprogramming the host microbiota. Meanwhile, a double-blind randomized placebo-controlled trial demonstrated that FMT significantly alters the recipient’s intestinal phage community and promotes the long-term stable colonization of donor phages (Zuppi et al., 2024). Therefore, we have incorporated FVT as a novel transplant intervention into this chapter. FVT has emerged as an innovative therapeutic strategy focused specifically on transferring viral components of the donor’s gut microbiota (Castells-Nobau et al., 2024). FVT involves transplanting the viral components present in feces, specifically bacteriophages (Rasmussen et al., 2020). Experiments by Ritz et al. (2024) demonstrate that FVT can alleviate stress-induced behavioral, immunological, and neurobiological changes by modulating the MGB axis. FVT significantly reduces the risk of pathogen transmission associated with FMT through physical filtration and processing while maintaining therapeutic efficacy. Compared to FMT, it represents a safer potential treatment strategy capable of accommodating donor variability (Rasmussen et al., 2024). At present, few studies have been conducted in this area, and more research is required to clarify the underlying mechanisms.

Although FMT and FVT are promising therapeutic approaches, their clinical application poses significant safety concerns. Regarding FMT, reports of serious adverse events, including the transmission of multidrug-resistant infections resulting in fatalities—have prompted regulatory warnings (Kuijper et al., 2019). The long-term consequences of introducing intact foreign microbial communities remain unclear, and adverse reactions such as diarrhea, bloating, and abdominal pain may occur. For FVT, although the risk of bacterial infection is significantly reduced during preparation by eliminating bacteria, the possibility of eukaryotic virome transmission and potential microbial contamination cannot be completely ruled out. As invasive therapeutic procedures, FMT and FVT require strict screening of microbiota donors and necessitate long-term follow-up in clinical trials. Furthermore, these therapies face significant regulatory hurdles. Neither FMT nor FVT fits neatly into traditional drug or medical device classifications, creating regulatory ambiguity. The absence of standardized protocols for donor screening, material preparation, and efficacy assessment complicates quality control and approval processes. Establishing safe, high-quality donor banks involves complex ethical and legal challenges. Crucially, current evidence supporting the efficacy of FMT/FVT for treating mood disorders primarily stems from small-scale or open-label studies, which are highly susceptible to bias and placebo effects. Before considering any clinical application recommendations, large-scale randomized double-blind placebo-controlled trials are imperative.

### 5.3 Dietary interventions

It is well known that diet significantly influences the composition of gut microbiota (Zou et al., 2021). Recent studies indicate that dietary factors can regulate depressive symptoms through both GM-dependent and -independent pathways (Maitiniyazi et al., 2022). Adherence to a healthy diet serves as an effective complementary strategy in managing depressive and anxiety disorders (Hamad et al., 2024; Enriquez-Martinez et al., 2021). Encouraging wholesome dietary practices should be integral to preventive and therapeutic approaches for depressive symptoms (Francis et al., 2022; Table 3). Some studies indicate that dietary diversity is inversely associated with symptoms of major depressive disorder (MDD) and generalized anxiety disorder (GAD), but reverse causality cannot be excluded (Pengpid and Peltzer, 2024). Diets associated with reduced anxiety and depression include “healthy” dietary patterns, the Mediterranean diet, anti-inflammatory diets, and diverse dietary practices (Lassale et al., 2019; Aucoin et al., 2021). In particular, the Mediterranean diet has demonstrated effectiveness for anxiety and depression (Johnson et al., 2022; Alnabulsi et al., 2024). This diet primarily includes olive oil as a fat source, abundant plant-based foods, fish, and limited intake of red meat, processed meat, and sweets (Bizzozero-Peroni et al., 2022; Altun et al., 2021). Such dietary patterns reduce oxidative stress and pro-inflammatory cell expression, both of which are key factors in depressive states (Bizzozero-Peroni et al., 2022; Fitó et al., 2007; Koelman et al., 2022; Huang et al., 2019). Multiple surveys examining populations from various countries, genders, and health conditions consistently support the beneficial role of the Mediterranean diet in anxiety and depression. Bizzozero-Peroni et al. (2025) recent meta-analysis indicates that the Mediterranean diet holds significant potential for alleviating depressive symptoms in individuals with depression. Tolkien et al. (2019) meta-analysis included 11 studies and concluded that adopting an anti-inflammatory diet may be an effective intervention or preventive measure for reducing the risk and symptoms of depression. Dong et al. (2024) team identified nonlinear bidirectional associations between dietary diversity and depressive symptoms through questionnaires and cross-lagged modeling analyses. Persistent inflammatory states are also linked to depression and anxiety (Flores Saiffe Farías et al., 2018). An analysis by Lv et al. (2022) further confirmed that anti-inflammatory dietary interventions could alleviate depressive symptoms in older adults. Additionally, the ketogenic diet may also improve anxiety or depression (Wu et al., 2022). Its neuroprotective and anti-inflammatory properties have been consistently demonstrated and may underline its antidepressant effects (Smolensky et al., 2023). Animal studies, such as those conducted by Gumus et al. (2022) showed that combining a ketogenic diet with voluntary exercise improves anxiety- and depression-like behaviors in mice, but larger-scale randomized clinical trials remain limited. Healthy dietary interventions can effectively reduce inflammation (Burrows et al., 2020). A recent observational study also showed that Dietary Approaches to Stop Hypertension (DASH) and the Mediterranean-DASH Intervention for Neurodegenerative Delay (MIND) diets have antidepressant potential (Cherian et al., 2021). Micronutrients, *ω*-3 polyunsaturated fatty acids, dietary fiber, polyphenols, and other bioactive dietary ingredients also show significant promise in improving depressive symptoms through different mechanisms (Wu et al., 2022).

**Table 3:** Summary of dietary interventions in anxiety and depression.

Dietary interventions	Key components	Mechanisms	Sample sizes (Examples)	Clinical outcomes	Reference
Mediterranean diet	Olive oil as a fat source, abundant plant-based foods, fish, and limited intake of red meat, processed meat, and sweets	Reduce oxidative stress and pro-inflammatory cell expression	PREDIDEP randomized trial; Dietary intervention group (*n* = 103), control group (*n* = 93)	Alleviate symptoms of depressive sub-syndrome	Cabrera-Suárez et al. (2024)
Anti-inflammatory diet	Promote foods with anti-inflammatory properties: berries, fish, leafy greens, and nuts; avoid pro-inflammatory foods such as sugar, refined carbohydrates, sweets, and processed meats	TNF-α and C-Reactive Protein (CRP) levels decreased significantly.	RCT; Intervention group (*n* = 35), Control group (*n* = 35)	Improve depressive symptoms	Cheng et al. (2025)
Ketogenic diet	Low-carbohydrate, high-fat, moderate-protein	Increase blood ketone levels	Preclinical; Intervention group (*n* = 16), Control group (*n* = 16)	The ketogenic diet combined with exercise can reduce anxiety and depressive behaviors	Gumus et al. (2022)
DASH diet	Rich in fruits, vegetables, whole grains, low-fat dairy	Reducing inflammation markers to mitigate the risk of depression	Prospective cohort study; 14,051 participants	The DASH diet is associated with a reduced risk of depression	Perez-Cornago et al. (2017)

### 5.4 Pharmacological and integrative approaches

#### 5.4.1 Conventional antidepressants and anxiolytics

Oral antidepressants and anxiolytics continue to dominate the treatment of anxiety and depression. SSRIs and SNRIs serve as primary medications for managing depression and anxiety, while benzodiazepines are restricted to short-term anxiety management (Neil-Sztramko et al., 2025). Antidepressants are generally categorized into novel antidepressants, tricyclic antidepressants, traditional antidepressants, and SSRIs, collectively offering various therapeutic options for depression treatment (Hockenberry et al., 2019). Studies have shown that antidepressants affect gut microbiota composition and function (Tan et al., 2022). SSRIs modulate gut microbiota, potentially contributing to symptom relief in depressed individuals. Individuals treated with SSRIs showed significant alterations in gut microbiota composition, which might explain improvements in mood symptoms (Gao M. et al., 2023). Fluoxetine, an SSRI antidepressant, exerts effects beyond emotional stabilization, particularly in pregnant and nursing women. This drug alters gut microbiota balance and fundamentally modifies the metabolic functions of these microorganisms (Ramsteijn et al., 2020). Venlafaxine exerts antidepressant effects by inhibiting serotonin and norepinephrine reuptake, regulating serotonin and glutamate levels in mouse models of depression, and influencing gut microbiota diversity, particularly affecting key bacterial genera such as *Blautia*, *Oscillibacter*, *Tyzzerella*, *Butyricoccus*, and *Enterorhabdus* (Shen et al., 2023). Dexipramine, a tricyclic antidepressant, exhibited notable antimicrobial activity; notably, *Mucinophilic Immobilized Bacillus* and *Escherichia coli* were highly susceptible to this drug (Ait Chait et al., 2020). Evidence suggests antidepressants can regulate gut microbiota composition and function, which may be critical for their efficacy. Future research exploring this interaction could inform improved treatment strategies.

#### 5.4.2 Traditional Chinese medicine

Currently, an increasing number of studies indicate that TCM treatments can alleviate psychological disorders, including depression and anxiety, by modulating the gut microbiota. The effectiveness of acupuncture (including electroacupuncture and manual acupuncture) in treating depression and anxiety has long been demonstrated (Zheng et al., 2019). Several studies show acupuncture can alleviate depression by modulating gut microbiota (Wang et al., 2024). Recent studies suggest acupuncture may ameliorate depressive-like behaviors in post-stroke depression (PSD) by modulating gut microbiota and inhibiting NLRP3 inflammasome overactivation in the colon (Cai et al., 2024). Electroacupuncture (EA) has minimal side effects, combining electrical stimulation and acupuncture. This innovative therapy has attracted attention in the field of neurological disorders, especially depression (Duan et al., 2024). EA may exert antidepressant effects by regulating gut microbiota (Duan et al., 2024), experimentally demonstrated by the upregulation of BDNF and increased abundance of SCFA-producing bacteria (Li S. et al., 2024). Umbilical moxibustion helps rebalance intestinal microbiota by promoting beneficial bacteria growth and inhibiting harmful strains. This microbial regulation plays an important role in alleviating subclinical depressive symptoms. The therapy creates a healthier intestinal environment, supporting mental health (Yu et al., 2024).

In addition, TCM herbal formulas have demonstrated efficacy in treating mental illnesses, including depression and anxiety, through alterations in gut microbiota (Table 4). Research indicates that Xiaoyao San alleviates depressive-like symptoms in rats subjected to chronic unpredictable mild stress (CUMS) by modulating gut microbiota, especially, it exerts antidepressant effects by inhibiting the TLR4/NLRP3 inflammatory pathway and enhancing intestinal barrier function (Liu et al., 2024). Similarly, data confirms that Gegen Qinlian decoction significantly alleviates depression-like behaviors in CUMS rats, also by regulating gut microbiota and metabolites such as oleanolic acid (Peng et al., 2024). Li J. L. et al. (2024) evaluated the efficacy and safety of Kaixin Powder in treating depression. The results showed that KXS was comparable to or superior to antidepressants in treating depression, with fewer side effects. KXS alleviates intestinal inflammation by modulating microbiota composition and reducing LPS levels, thereby decreasing pro-inflammatory cytokines and protecting both the intestinal barrier and blood–brain barrier. Simultaneously, it lowers HPA axis hormone levels to mitigate excessive HPA axis activation, thereby improving anxiety and depression (Cao et al., 2020). Dan Zhi Xiao Yao San exerts its antidepressant effects through a multi-component, multi-target, multi-pathway mechanism. Its primary active constituents are quercetin and luteolin, with key targets including AVPR2, EGFR, F2, and CDK6. Therapeutic effects are achieved by regulating LPA and the gut microbiota-brain axis (Zhu X. et al., 2024). Zhi-zi-chi decoction not only regulates intestinal microbiota but also delivers multiple bioactive compounds (such as oleanolic acid) that act on multi-target genes. This ultimately modulates the HPA axis (by reducing CRH, ACTH, etc.), elevates neurotransmitter levels (5-HT, GABA, etc.), reduce pro-inflammatory factors (IL-1β, TNF-*α*), and increase anti-inflammatory factors (IL-10) to alleviate anxiety and depression (Tian et al., 2024). In summary, robust evidence supports the efficacy of TCM interventions in alleviating depression and anxiety symptoms through gut microbiota regulation.

**Table 4:** Effects of herbal formulas on the composition of the intestinal microbiota.

Chinese medicine formula	Herb composition	Subject	Antidepressant mechanisms	Microbiota changes	Conclusion	Reference
Danzhi-Xiaoyao-San(DZXYS)	Baizhu, Chaihu, Danggui, Fuling, Gancao, Danpi, Zhizi, Baishao	depressed patients	lysophosphatidic acid (LPA); MGB axis	*Ascomycetes*, *Actinobacteria*, *Verrucomicrobia↑*, *Firmicutes, Bacteroidetes↓*	DZXYS in combination with SSRIs improves depression more effectively than SSRIs alone	Zhu et al. (2024)
Gegen Qinlian decoction(GGQLD)	Gegen, Huangqin, Huanglian, Gancao	CUMS rats	NA	*Bacteroides*, *Pseudomonas↑*, *Ruminococcus↓*	GGQLD alleviates depressive symptoms in CUMS rats	Peng et al. (2024)
Kai-Xin-San (KXS)	Renshen, Yuanzhi, Shichangpu, Fuling,	CUMS Mice	MGB axis; HPA axis	*Bifidobacteria, Allobaculum↑*, *Coprococcus*; *Helicobacter*, *Mucispirillum↓*	KXS improves depression-like behavior in CUMS mice	Cao et al. (2020)
Xiaoyaosan (XYS)	Baizhu, Chaihu, Danggui, Fuling, Gancao, Baishao	CUMS rats	MGB axis; HPA axis	*Lactobacillus*, *Adlercreutzia↑*, *Bacteroides*, *Corynebacterium↓*	XYS fights depression through the microbiota-gut-brain axis	Liu et al. (2024)
Zhi-zi-chi decoction (ZZCD)	Gardeniae Fructus, Semen Sojae Praeparatu	CUMS rats	MGB axis; HPA axis	*Corynebacterium, Allobaculum, Lactobacillus↑*, *Parabacteroides, Bilophila, Fusobacterium*, *Desulfovibrio↓*	ZZCD exerts antidepressant effects through microbiota-gut-brain regulation	Tian et al. (2024) and Gao F. Y. et al. (2023)

NA, represents no data.

However, translating these promising research findings into evidence-based medicine faces significant challenges, primarily due to the lack of standardization in traditional Chinese medicine formulations, which leads to substantial heterogeneity across studies. Future research must prioritize identifying key active components and developing standardized, quality-controlled TCM formulations to ensure reproducibility and reliability of results. Furthermore, experimental findings from animal models cannot be directly applied to human clinical settings due to species-specific differences in gut microbiota composition, host metabolism, and drug pharmacokinetics. Animal experiments can only partially replicate the complexity of human emotional disorders. Therefore, while our data provides strong mechanistic evidence, they cannot directly predict human efficacy. Future research should integrate systems biology approaches to elucidate the precise mechanisms of action and active components of TCM. In summary, rigorously designed human clinical trials using standardized TCM formulations are essential for validating their efficacy and safety in treating anxiety and depression.

## 6 Discussion

### 6.1 Limitations

Overall, the relationship between gut microbiota and anxiety and depression described in this review has been confirmed by numerous experiments, however, the study still has limitations. While preclinical models have been invaluable for elucidating mechanistic pathways along the gut-brain axis, current studies primarily involve animal models. Animal models cannot fully replicate the complexity of human mental disorders, social stressors, and dietary habits. Moreover, there are species differences between the animal gut microbiota and humans, and variations also exist among gut microbiota across different human populations. Although animal study results appear promising, translating these findings into human clinical applications remains challenging. Currently, there is a relative scarcity of high-quality, large-scale randomized controlled trials (RCTs) in humans, and animal studies currently dominate. Much current research in this field does not adequately consider subjects’ genetic predisposition, environmental factors, medication history, and dietary habits, all of which profoundly influence gut microbiota composition (Xie et al., 2024). Studies have shown substantial variability in gut microbiota composition among individuals with depression. This heterogeneity may result from differences in biological characteristics among study cohorts and methodological variations in sample processing (Averina et al., 2024). This likely explains the inconsistent efficacy observed in clinical trials involving probiotics, prebiotics, or fecal microbiota transplantation—where a particular intervention may prove effective in one group but ineffective in another. Significant methodological differences between studies constitute a key obstacle to progress in this field. Variations in bioinformatics analyses, intervention formulations (such as probiotic strains, dosages, and treatment durations), and the lack of standardized outcome measures for gastrointestinal and psychological symptoms hinder meta-analyses and cross-study comparisons. Furthermore, many studies are constrained by small sample sizes, resulting in insufficient analytical power and increased risk of false-positive findings. Each intervention type has unique risk–benefit characteristics and should thus be applied appropriately according to specific contexts. The main limitation of probiotics lies in their strain-specific effects; FMT has unknown long-term safety implications; and TCM faces challenges related to standardization. These factors pose significant challenges in clinical trials related to gut microbiota.

### 6.2 Future directions

Therefore, future studies should involve broader and more diverse populations to verify the generalizability and therapeutic potential of these treatments. Meanwhile, future research should explore the impact of specific microorganisms and their by-products on mental health, particularly their role in emotional regulation. Our findings underscore the imperative to prioritize rigorous human RCTs to validate efficacy and ensure safety for the target patient population. Future research must adopt consensus methodologies and standardize core outcome measures to enhance reproducibility and data integration capabilities. The field of psychiatry requires a shift toward personalized microbiome-targeted interventions, where treatment plans are tailored based on an individual’s baseline microbial composition, metabolome profile, and clinical characteristics. For instance, patients exhibiting specific inflammatory microbial signatures may benefit from distinct bacterial strains, while those characterized by insufficient SCFAs production require different strains. To achieve personalized treatment, integrating multi-omics approaches is crucial. By integrating metagenomics, metabolomics, and proteomics, we can comprehensively unravel disease mechanisms and the interactions between the microbiome and the host. This multi-omics approach also enables the discovery of biomarkers, advancing precision medicine and ultimately guiding the development of novel diagnostic methods and targeted therapeutic agents. Additionally, researchers need to clarify more precisely how probiotics, prebiotics, and other interventions influence specific neurobiological pathways. Current studies largely lack long-term follow-up, leaving treatments without sufficient experimental data to assess long-term efficacy and potential side effects. Addressing these gaps through comprehensive analysis could facilitate the development of more diverse and effective therapeutic strategies for anxiety and depression in the future.
