# Supplementary material for: Gut microbiota as a novel target for treating anxiety and depression: from mechanisms to multimodal interventions
Source: Front Microbiol. 2025 Oct 21;16:1664800. doi: 10.3389/fmicb.2025.1664800 (PMC12585160; doi:10.3389/fmicb.2025.1664800)
Supplement: Supplementary file 1 [file Table_1.DOCX]

| Table S1. Search strategy | |
| --- | --- |
| No. | Search items |
| #1 | "Gastrointestinal Microbiome"[Mesh] |
| #2 | (((((((((((((((((((((((((((((((((((((((((((((((((((((((((Gastrointestinal Microbiome[Title/Abstract]) OR (Gastrointestinal Microbiomes[Title/Abstract])) OR (Microbiome, Gastrointestinal[Title/Abstract])) OR (Microflora, GI[Title/Abstract])) OR (GI Microflora[Title/Abstract])) OR (GI Microfloras[Title/Abstract])) OR (Microfloras, GI[Title/Abstract])) OR (Microbiome, GI[Title/Abstract])) OR (GI Microbiome[Title/Abstract])) OR (GI Microbiomes[Title/Abstract])) OR (Microbiomes, GI[Title/Abstract])) OR (Enteric Microbiota[Title/Abstract])) OR (Enteric Microbiotas[Title/Abstract])) OR (Microbiota, Enteric[Title/Abstract])) OR (Microbiotas, Enteric[Title/Abstract])) OR (Flora, Enteric Microflora[Title/Abstract])) OR (Enteric Microflora Flora[Title/Abstract])) OR (Enteric Microflora Floras[Title/Abstract])) OR (Floras, Enteric Microflora[Title/Abstract])) OR (Microflora Flora, Enteric[Title/Abstract])) OR (Microflora Floras, Enteric[Title/Abstract])) OR (Gut Microflora[Title/Abstract])) OR (Microflora, Gut[Title/Abstract])) OR (Gastrointestinal Microflora[Title/Abstract])) OR (Microflora, Gastrointestinal[Title/Abstract])) OR (Gastrointestinal Flora[Title/Abstract])) OR (Flora, Gastrointestinal[Title/Abstract])) OR (Gut Flora[Title/Abstract])) OR (Flora, Gut[Title/Abstract])) OR (Gastrointestinal Microbial Community[Title/Abstract])) OR (Gastrointestinal Microbial Communities[Title/Abstract])) OR (Microbial Community, Gastrointestinal[Title/Abstract])) OR (Gut Microbiome[Title/Abstract])) OR (Gut Microbiomes[Title/Abstract])) OR (Microbiome, Gut[Title/Abstract])) OR (Gastrointestinal Microbiota[Title/Abstract])) OR (Gastrointestinal Microbiotas[Title/Abstract])) OR (Microbiota, Gastrointestinal[Title/Abstract])) OR (Microflora[Title/Abstract])) OR (Microfloras[Title/Abstract])) OR (Gut Microbiota[Title/Abstract])) OR (Gut Microbiotas[Title/Abstract])) OR (Microbiota, Gut[Title/Abstract])) OR (Intestinal Microbiome[Title/Abstract])) OR (Intestinal Microbiomes[Title/Abstract])) OR (Microbiome, Intestinal[Title/Abstract])) OR (Intestinal Microflora[Title/Abstract])) OR (Microflora, Intestinal[Title/Abstract])) OR (Intestinal Flora[Title/Abstract])) OR (Flora, Intestinal[Title/Abstract])) OR (Intestinal Microbiota[Title/Abstract])) OR (Intestinal Microbiotas[Title/Abstract])) OR (Microbiota, Intestinal[Title/Abstract])) OR (Enteric Bacteria[Title/Abstract])) OR (Bacteria, Enteric[Title/Abstract])) OR (Gastric Microbiome[Title/Abstract])) OR (Gastric Microbiomes[Title/Abstract])) OR (Microbiome, Gastric[Title/Abstract]) |
| #3 | #1 OR #2 |
| #4 | "Depression"[Mesh] |
| #5 | (((((Depression[Title/Abstract]) OR (Depressive Symptoms[Title/Abstract])) OR (Depressive Symptom[Title/Abstract])) OR (Symptom, Depressive[Title/Abstract])) OR (Emotional Depression[Title/Abstract])) OR (Depression, Emotional[Title/Abstract]) |
| #6 | #4 OR #5 |
| #7 | "Anxiety"[Mesh] |
| #8 | ((((((((Anxiety[Title/Abstract]) OR (Angst[Title/Abstract])) OR (Nervousness[Title/Abstract])) OR (Hypervigilance[Title/Abstract])) OR (Social Anxiety[Title/Abstract])) OR (Anxieties, Social[Title/Abstract])) OR (Anxiety, Social[Title/Abstract])) OR (Social Anxieties[Title/Abstract])) OR (Anxiousness[Title/Abstract]) |
| #9 | #8 OR #7 |
| #10 | #6 OR #9 |
| #11 | "Brain-Gut Axis"[Mesh] |
| #12 | (((((((((((((((((((((((((Brain-Gut Axis[Title/Abstract]) OR (Axis, Brain-Gut[Title/Abstract])) OR (Brain Gut Axis[Title/Abstract])) OR (Brain[Title/Abstract] AND Gut Axis[Title/Abstract])) OR (Gut[Title/Abstract] AND Brain Axis[Title/Abstract])) OR (Gut-Brain Axis[Title/Abstract])) OR (Axis, Gut-Brain[Title/Abstract])) OR (Gut Brain Axis[Title/Abstract])) OR (Microbiota-Gut-Brain Axis[Title/Abstract])) OR (Axis, Microbiota-Gut-Brain[Title/Abstract])) OR (Microbiota Gut Brain Axis[Title/Abstract])) OR (Microbiome-Brain-Gut Axis[Title/Abstract])) OR (Axis, Microbiome-Brain-Gut[Title/Abstract])) OR (Microbiome Brain Gut Axis[Title/Abstract])) OR (Brain-Gut-Microbiome Axis[Title/Abstract])) OR (Axis, Brain-Gut-Microbiome[Title/Abstract])) OR (Brain Gut Microbiome Axis[Title/Abstract])) OR (Microbiota-Brain-Gut Axis[Title/Abstract])) OR (Axis, Microbiota-Brain-Gut[Title/Abstract])) OR (Microbiota Brain Gut Axis[Title/Abstract])) OR (Gut-Brain-Microbiome Axis[Title/Abstract])) OR (Axis, Gut-Brain-Microbiome[Title/Abstract])) OR (Gut Brain Microbiome Axis[Title/Abstract])) OR (Microbiome-Gut-Brain Axis[Title/Abstract])) OR (Axis, Microbiome-Gut-Brain[Title/Abstract])) OR (Microbiome Gut Brain Axis[Title/Abstract]) |
| #13 | #11 OR #12 |
| #14 | #3 AND #10 AND #13 |
